# Repeat Cytology and Human Papillomavirus Screening Strategies in Detecting Preinvasive Cervical Lesions

**DOI:** 10.1097/MD.0000000000000435

**Published:** 2015-02-06

**Authors:** Kemin Li, Rutie Yin

**Affiliations:** From the Department of Gynaecology, West China Second University Hospital (KL, RY), Sichuan University, Chengdu, Sichuan, China

## Abstract

The aim of the present study was to determine the value of human papillomavirus (HPV) testing in screening patients with preinvasive cervical lesions.

Seven hundred thirty-four women diagnosed with atypical squamous cells of undetermined significance (ASCUS+) cervical cytology during routine screening had additional cytologic testing and HPV DNA testing within 6 months of their diagnosis, after which all women who tested positive were referred for colposcopy and biopsy. The test findings were then used to determine the screening value of HPV for diagnosing preinvasive cervical lesions.

Cytology and HPV testing were compared by conventional cytology. The odds ratio (OR) of sensitivity using ASCUS+ or low-grade squamous intraepithelial neoplasia (LSIL+) as a cutoff for detecting cervical intraepithelial neoplasia (CIN) II+ was, respectively, 0.78 (0.72, 0.85) and 0.82 (0.70, 0.95) (*P* < 0.01). The cytology for triage and conventional cytology had different sensitivities using ASCUS+ or LSIL+ as the cutoff (*P* < 0.01). The cytology or HPV testing and conventional cytology had a difference in sensitivity using ASCUS+, LSIL+, or high-grade squamous intraepithelial neoplasia (HSIL+) as the cutoff (*P* < 0.01). Cytology and HPV testing were also compared with conventional cytology. The OR of specificity using ASCUS+ or LSIL+ as the cutoff for the detection of CIN II+ was 1.97 (1.68, 2.31) and 1.10 (1.02, 1.18), respectively (*P* < 0.01). The cytology for triage and conventional cytology had a difference in specificity when ASCUS+ or LSIL+ was used as the cutoff (*P* < 0.01). Finally, the cytology or HPV testing and conventional cytology had a difference in specificity when ASCUS+, LSIL+, or HSIL+ was used as the cutoff (*P* < 0.01).

Cytology and HPV testing and cytology for triage improved the specificity of detecting CIN II+, but this did not improve the sensitivity. Additionally, cytology or HPV testing improved the sensitivity of detecting CIN II+ but not the specificity.

## INTRODUCTION

Worldwide, cervical cancer is the third most common cancer in women.^1^ Furthermore, regional data from China's National Cancer Registry indicated that 5473 new cervical cancer cases were reported. Moreover, the incidence of cervical cancer was 12.96/10 million in 2009. Finally, a total of 1387 deaths were reported, and the mortality associated with this cancer is 3.28/10 million.^2^

Persistent human papillomavirus (HPV) infection is the most important factor in the development of cervical cancer.^3,4^ This is especially relevant to China, as the HPV infection rate in Chinese women (both urban and rural women are taken into account) has been reported to be about 15%, the highest in the world.^5,6^ Additionally, a meta-analysis has revealed that persistent HPV infection is a major risk factor for the development of cervical cancer in Chinese married women.^7^

Cytologic screening majorly impacts the incidence and death rate of cervical cancer. Moreover, high-grade cervical intraepithelial neoplasia (CIN) is found in 5% to 17% of women following a diagnosis of low-grade CIN.^8–10^ Additionally, follow-ups given to patients previously diagnosed with either atypical squamous cells of undetermined significance (ASCUS) or low-grade squamous intraepithelial neoplasia (LSIL) revealed that 39% to 69% developed high-grade squamous intraepithelial neoplasia (HSIL). HPV DNA testing combined with cytology identifies more patients with cervical neoplasia or cervical cancer.^11–14^ The introduction of cervical screening reduced the mortality in China from 10.7/10 million in the 1970s to 3.89/10 million in the 1990s. Accordingly, cervical cancer screening (the main strategies include cytology for triage testing and cytology) has been included in the major public health services that are provided by the Chinese government. Nevertheless, the incidence of cervical cancer is actually increasing.^15^ The purpose of this study is to determine the value of HPV screening in patients with ASCUS.

## MATERIALS AND METHODS

Cervical erosion is a common problem. We identified outpatients who had been diagnosed with ASCUS or worse via cervical cytology screening from January 2010 to August 2013. Patients were excluded if they met any of the following conditions: they did not have a cervix, had a previous CIN diagnosis, or had a previous destructive cervical treatment. Patients were evaluated with ASCUS or with an additional Pap smear and HPV testing within 6 months. Patients with HSIL or carcinoma in situ (CIS) underwent colposcopies and biopsies and were treated based on their individual test results. These patients were not included in the study. This is a retrospective analysis, so the ethical approval was not necessary, and the study does not involve patient consent.

The cervical smear is obtained using a modified Ayre spatula with an elongated tip designed for a Pap smear. A cell sample is collected from the cervical os and transformation zone by swabbing. Then, the swab is placed into transport tubes that contain the appropriate medium for HPV testing. Our hospital's pathological medical research laboratories processed the repeat Pap smears, and then, the pathologists classified them according to the 2001 Bethesda System.^16^ Specimens with ASCUS or worse (ASCUS+) were classified as positive. The pathologists were not informed of the HPV test results and patient clinical information.

Additionally, Hybrid Capture II (HCII) was used to analyze the HPV DNA. Testing for high-risk HPV types 16, 18, 31, 33, 35, 39, 45, 51, 52, 56, 58, 59, and 68 was performed following the manufacturer's recommendations specimens were considered positive if the ratio of relative lights units (RLUs) of the specimen to the mean RLU of positive control triplicates was at least 1 equivalent to 1 pg of HPV DNA per milliliter. As before, the laboratory was not informed of the Pap smear results and patient clinical information.

All patients with positive conventional cytology (Pap smear) or HPV DNA testing had a colposcopy and cervical biopsy. The colposcopic examinations were performed at the West China Second Hospital of Sichuan University, Chengdu.

The Department of Pathology at the West China Second Hospital of Sichuan University processed the tissue specimens taken by the colposcopy clinic. Two gynecologic pathologists independently reviewed all of the specimens. For any diagnostic disagreements, the pathologists reviewed the findings together and then reached a final consensus. Additionally, the gynecologic pathologists were unknowledgeable of the HPV test results, Pap smear results, and the patient information. The histopathological results were reported^17^ as CIN I or worse (CIN I+), CIN II or worse (CIN II+), or CIN III or worse (CIN III+).

The following different screening strategies were evaluated:

1. Conventional cytology (Pap smear) alone: Specimens with ASCUS or worse were classified as positive.
2. HPV testing alone: Specimens with more than 1.0 pg/mL were classified as positive.
3. Conventional cytology (Pap smear) and HPV testing: Specimens with both ASCUS or worse (ASCUS+) and more than 1.0 pg/mL were classified as positive.
4. Cytology for triage: positive HPV referred for cytology and if ASCUS + are then referred for colposcopy.
5. Convention cytology (Pap smear) or HPV testing: Specimens with either ASCUS or worse (ASCUS+) or more than 1.0 pg/mL were classified as positive.

The sensitivity, specificity, and relative risk (RR) of the different screening strategies were calculated. The SAS 9.2 software was used. A χ^2^ test or Fisher exact test was used to compare categorical variables. A Student *t* test was used to evaluate continuous variables, and the K statistic was used to check for agreement. Exact 95% confidence intervals (CIs) were calculated. All *P* values were 2-sided. *P* < 0.01 was considered statistically significant.

## RESULTS

Seven hundred thirty-four women (21–66 years old) with ASCUS or worse on a cervical cytology screening were identified. In Table 1, the sensitivities and specificities of CIN screening strategies are shown. Additionally, the RRs of sensitivity and specificity in the screening strategies for CIN I+, CIN II+, and CIN III+ are, respectively, shown in Tables 2–4. Finally, the positive predictive values (PPVs) of every screening strategy are shown in Table 5.

**Table 1:**
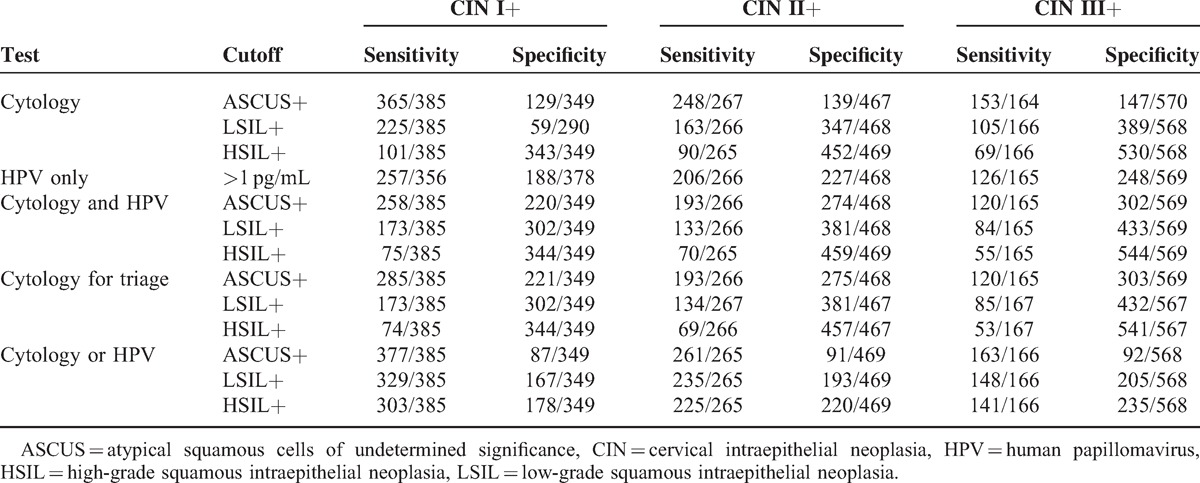
Sensitivity and Specificity of Screening Strategies for CIN

**Table 2:**
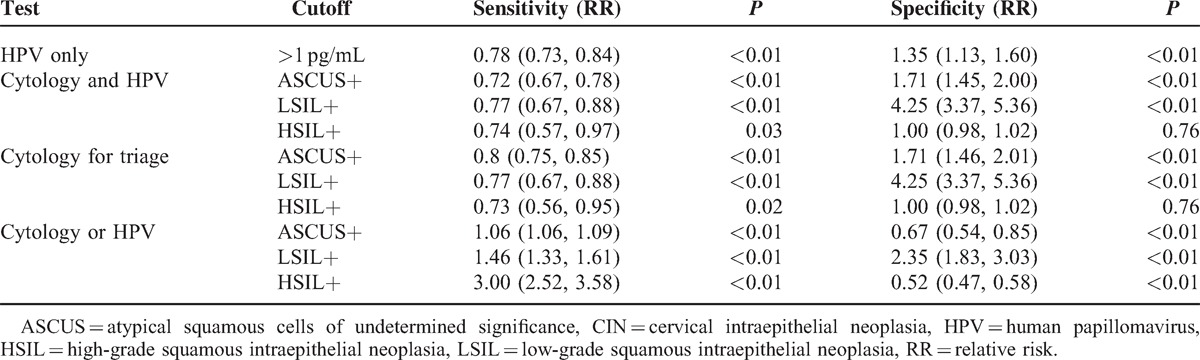
RR of Sensitivity and Specificity in Screening Strategies for CIN I+

**Table 3:**
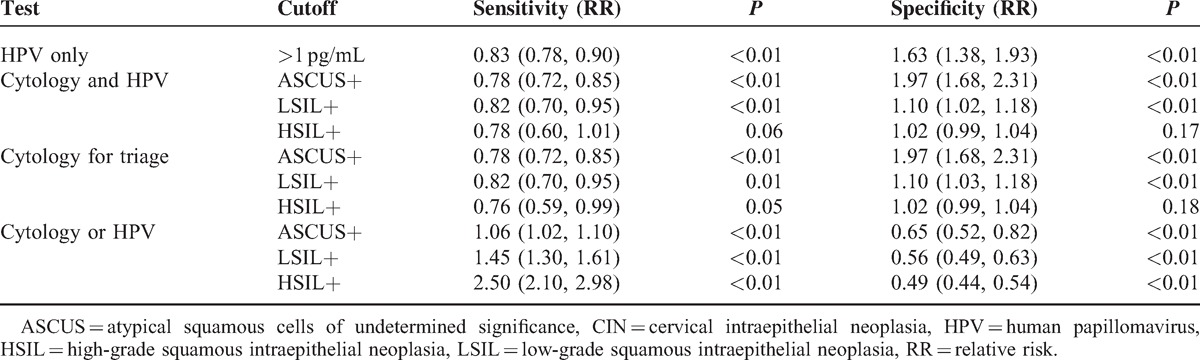
RR of Sensitivity and Specificity in Screening Strategies for CIN II+

**Table 4:**
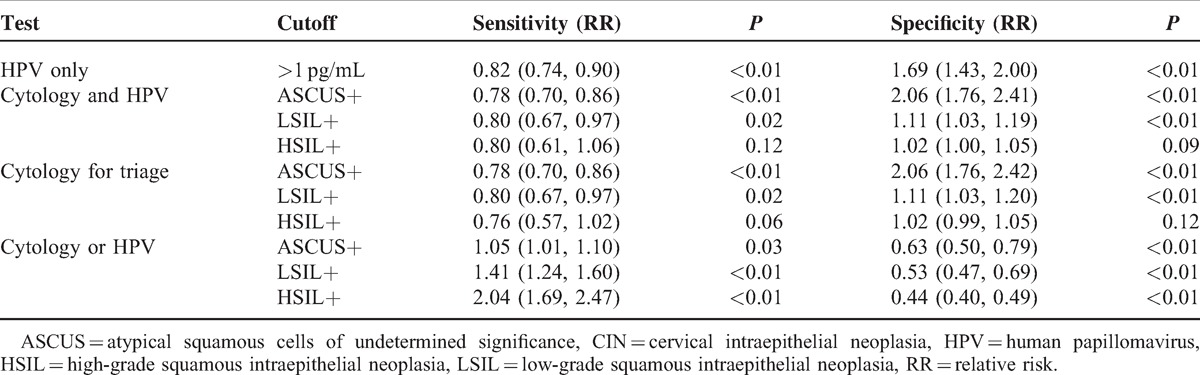
RR of Sensitivity and Specificity in Screening Strategies for CIN III+

**Table 5:**
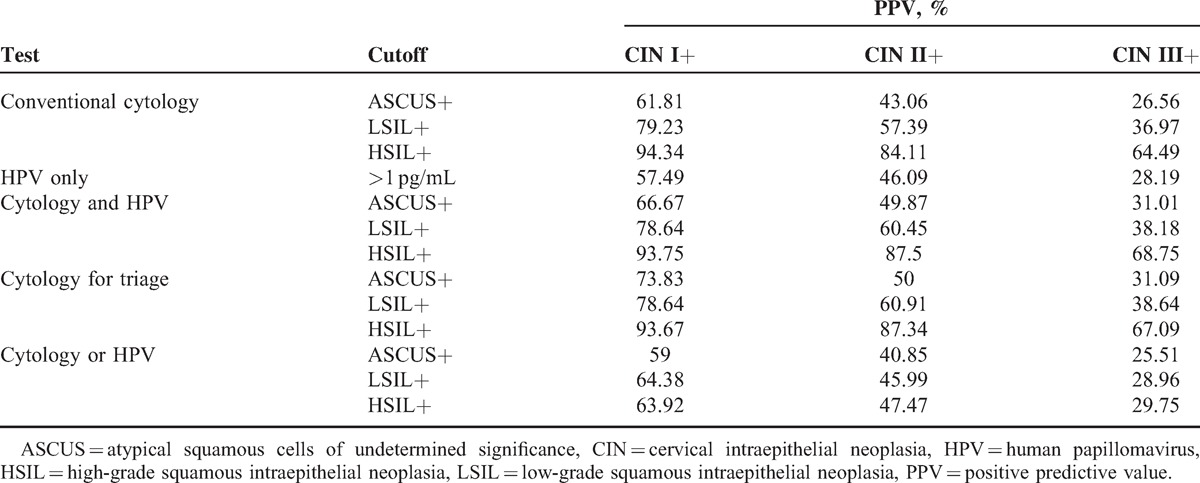
PPV of Strategies Used to Identify CIN

### Strategies for Screening CIN II+

The RRs of sensitivity and specificity in the screening strategies for CIN II+ are shown in Table 3.

#### HPV-Only Testing Versus Conventional Cytology

The RR of sensitivity using ASCUS+ as a cutoff for detecting CIN II+ was 0.83 (0.78, 0.90) (*P* < 0.01). There was also a difference in specificity using ASCUS+ as a cutoff.

#### Conventional Cytology and HPV Testing Versus Conventional Cytology

The RR of sensitivity using ASCUS+ or LSIL+ as a cutoff for detecting CIN II+ was 0.78 (0.72, 0.85) and 0.82 (0.70, 0.95), respectively (*P* < 0.01). There was no difference in sensitivity using HSIL+ as a cutoff; however, there was a difference in specificity using ASCUS+ or LSIL+ as a cutoff (*P* < 0.01). There was no difference in specificity using HSIL+ as a cutoff.

#### Conventional Cytology for Triage Testing Versus Conventional Cytology

The RR of sensitivity using ASCUS+ as a cutoff for detecting CIN II+ was 0.78 (0.72, 0.85) (*P* < 0.01). There was no difference in sensitivity using LSIL+ or HSIL+ as a cutoff; however, there was a difference in specificity using ASCUS+ or LSIL+ as a cutoff (*P* < 0.01). There was no difference in specificity using HSIL+ as a cutoff.

#### Convention Cytology or HPV Testing Versus Conventional Cytology

The RR of sensitivity using ASCUS+, LSIL+, or HSIL+ as a cutoff for detecting CIN II+ was 1.06 (1.02, 1.10), 1.45 (1.30, 1.61), and 2.50 (2.10, 2.98), respectively (*P* < 0.01). There was a difference in specificity using ASCUS+, LSIL+, or HSIL+ as the cutoff (*P* < 0.01).

### Strategies for Screening CIN III+

The RRs of sensitivity and specificity in screening strategies for CIN III+ are shown in Table 4.

#### HPV-Only Testing Versus Conventional Cytology

The RR of sensitivity using ASCUS+ as a cutoff for detecting CIN III+ was 0.82 (0.74, 0.90) (*P* < 0.01). There was a difference in specificity using ASCUS+ as a cutoff.

#### Conventional Cytology and HPV Testing Versus Conventional Cytology

The RR of sensitivity using ASCUS+ as a cutoff for detecting CIN III+ was 0.78 (0.70, 0.86) (*P* < 0.01). There was no difference in sensitivity using LSIL+ or HSIL+ as the cutoff; however, there was a difference in specificity using ASCUS+ or LSIL+ as the cutoff (*P* < 0.01). There was no difference in specificity using HSIL+ as the cutoff.

#### Conventional Cytology for Triage Testing Versus Conventional Cytology

The RR of sensitivity using ASCUS+ as a cutoff for detecting CIN III+ was 0.78 (0.70, 0.86) (*P* < 0.01). There was no difference in sensitivity using LSIL+ or HSIL+ as the cutoff, although there was a difference in specificity using ASCUS+ or LSIL+ as the cutoff (*P* < 0.01). There was no difference in specificity using HSIL+ as the cutoff.

#### Conventional Cytology or HPV Testing Versus Conventional Cytology

The RR of sensitivity using LSIL+ or HSIL+ as the cutoff for detecting CIN III+ was 1.41 (1.24, 1.60) and 2.04 (1.69, 2.47), respectively (*P* < 0.01). There was no difference in sensitivity using ASCUS+ as the cutoff (*P* > 0.01), but there was a difference in specificity using ASCUS+, LSIL+, or HSIL+ as the cutoff (*P* < 0.01).

## DISCUSSION

HPV is a sexually transmitted virus that is associated with lesions in the anogenital tract.^18^ More specifically, high-risk subtypes (16, 18, 31, 33, 35, 39, 45, 51, 52, 56, 58, 59, and 68) have been associated with cervical cancer. However, HPV testing is a new technique to diagnose cervical dysplasia. Several HPV testing strategies have been used including HPV-only testing, HPV as the primary test, and HPV combined with cytology. The NCCN guideline V.2.2012 recommends that HPV DNA testing should not be used alone for screening or as a means of replacing other cervical cancer screening methods. However, the Food And Drug Administration has approved high-risk HPV DNA testing as an adjunct to cervical cytologic testing. Using a combination of different tests may, accordingly, identify more patients with cervical neoplasia.^19^

Seven hundred thirty-four women, 21 to 66 years old, diagnosed with ASCUS or worse found on their screening cervical cytologies were evaluated with repeat conventional cytology, HPV DNA testing, colposcopy, and cervical biopsy. Additionally, on being initially diagnosed with ASCUS, some patients received a colposcopy and were diagnosed as high-grade CIN; these patients were excluded from the analysis. The ability of 4 different screening methods (HPV-only testing, conventional cytology and HPV testing, conventional cytology for triage, and conventional cytology or HPV testing) to identify preinvasive cervical lesions was evaluated. Three different cutoffs—ASCUS+, LSIL+, and HSIL+—were used to test these strategies. Three screening strategies (HPV-only testing, conventional cytology and HPV testing, conventional cytology for triage) improved the specificity of screening for preinvasive cervical lesions, but these strategies did not improve the sensitivity. More specifically, conventional cytology or HPV testing improved the sensitivity of screening for preinvasive cervical lesions, but neither improved the specificity. The PPVs of the 2 strategies for screening CIN II+ or CIN III+ using ASCUS+, LSIL+, or HSIL+ as a cutoff were better than either of the other 2 strategies.

Moreover, a disadvantage of combined testing is that it is more costly; however, this cost is counterbalanced by the combined testing's ability to detect two-thirds more cases of CIN than Pap tests alone.^14^ Additionally, China has an unbalanced development of urban and rural medical technology and is considered a developing country. Combined testing, including conventional cytology for triage and convention cytology and HPV testing, is recommended in developed urban areas. However, conventional cytology may be more appropriate in rural areas. The present study did not address the costs of HPV testing and colposcopies in China, so this conclusion needs to be further studied.

Finally, it is well known that HPV testing has quite a high sensitivity—higher than cytology—but continues to have a low specificity. However, this study determined that 3 screening strategies (HPV-only testing, conventional cytology and HPV testing, and conventional cytology for triage) improved the specificity of screening for preinvasive cervical lesions but did not improve the sensitivity. This inconsistency may be explained by a small sample size. Moreover, cytology with HPV testing diagnosed more patients with cervical neoplasia. HPV testing in our patients improved the sensitivity of cervical cancer screening but not the specificity. However, some of the other findings were inconsistent. Large, randomized controlled studies are needed to better evaluate these findings.
